# Commentary on Sun and Tang: Measurement assessment and validity in problematic smartphone use

**DOI:** 10.1111/add.16764

**Published:** 2025-01-11

**Authors:** Richard J. E. James, Lucy Hitcham

**Affiliations:** ^1^ School of Psychology University of Nottingham Nottingham UK; ^2^ School of Medicine University of Nottingham Nottingham UK; ^3^ Horizon Centre for Doctoral Training University of Nottingham Nottingham UK

**Keywords:** measurement, problematic smartphone use, psychometrics, scale development, smartphone addiction, smartphone use

*Digital technologies have been targeted for restrictions, partly based on their purported addictive tendencies. However, the field is plagued by measurement problems. This commentary explores some of the key lessons drawn from Sun and Tang's study, and the implications for measuring both problematic smartphone use and other addictive behaviours*.

Intense debate about the impact of new technologies such as smartphones, particularly on children and adolescents, has led to proposals to ban, limit access to or restrict content on devices and platforms [1, 2]. One of the concerns fuelling this debate is whether these technologies are addictive. However, there are serious, fundamental problems with how ‘addiction’ is measured and conceptualized in these contexts [3, 4]. Sun and Tang [5] present findings from a large‐scale revision and re‐validation of the widely used Smartphone Addiction Scale for Chinese students (SAS‐C) [6]. The authors respond to existing conceptual critiques of smartphone addiction by reorienting the scale around problematic smartphone use (PSU) and assessing measurement invariance of the revised scale across sex, type of university and resident city. This level of comprehensive assessment is often lacking in studies looking to validate smartphone or other behavioral addiction scales.

The thoughtful choice of estimation procedures for the confirmatory factor analysis (CFA) and invariance testing is worth particular attention. Many assessment studies use maximum likelihood (ML or MLR with robust standard errors) for CFA despite well‐known limitations when applied to ordinal data [7]. A popular alternative is to use limited information estimation, for example, weighted least squares (WLSMV) to overcome these. However, doing so comes with major drawbacks, most notably when assessing measurement invariance [8, 9]. Sun and Tang [5] carefully balance the strengths of both MLR and WLSMV to validate the Problematic Smartphone Use Scale among Chinese college students (PSUS‐C). These considerations are valuable across the entirety of addiction research, especially in domains or populations where endorsement of indicators might be skewed (e.g. gambling, certain forms of substance use and general population samples). To illustrate why these problems matter, CFA studies have repeatedly shown inconsistent evidence of structural validity in prominent scales such as the Problem Gambling Severity Index [10, 11]. However, closer examination suggests that most of this inconsistency is an artifact of using ML on ordinal questionnaire items in general population samples where the distribution of responses is often skewed. When analyzed using an approach that balances the strengths of both ML and WLSMV, these inconsistencies disappear [11, 12].

The findings also highlight an important tension between identifying the best‐fitting factor structure and deciding how a scale should be used. Both exploratory factor analysis (EFA) and CFA rejected a single‐factor model in this study, yet a sum score was used to assess criterion validity. We raise this to promote the benefits of testing models specifying either a second‐order or a bifactor structure because these can assess whether a single score is appropriate [13]. This is an issue across the PSU field, where many scales have been validated as multi‐dimensional. but are used as a single score. This tension is a source of analytic flexibility and a potential threat to the validity of many findings, especially when methods such as structural equation modelling are used.

Our final reflection underscores the importance of invariance testing. Despite concluding in favor of strict invariance, there does not appear to be a comparison of latent mean differences that would allow a stronger test of group differences. Our examination of the descriptive data suggests the absence of a substantial sex difference in PSUS‐C scores in this large, externally representative sample. We calculated the standardized effect size (d) using the mean (M) and SD statistics reported in table 1 (men: M = 58.05, SD = 18.09; women: M = 57.52, SD = 16.12). The difference observed in this study does not appear to practically differ from zero (d = 0.03). This finding contrasts with a large, disparate literature that has inconsistently found sex differences in the severity and prevalence of problematic smartphone behaviors (e.g. Cohen's *d* for women > men = 0.16 [14], 0.39 [15], 0.22 [16], 0.10 [17] and 0.21 [17]). This is further complicated by a fixation on creating novel instruments or adapting scales from other behavioral addictions instead of improving and refining existing measures [18]. Ultimately, the absence of appropriate psychometric validation found in many PSU and behavioral addiction measures makes it impossible to determine whether the group differences observed elsewhere reflect genuine differences or bias caused by sampling, specific measurement scales or specific questionnaire items. Sun and Tang's study [5] offers insights on how to move forward with the assessment and validation of behavioral addiction measurement scales. The use of rigorous testing is essential to establish whether addiction constructs are equivalent across diverse groups of people to make valid group comparisons and inferences [19].

## AUTHOR CONTRIBUTIONS

**Richard J. E. James:** Writing—original draft (equal). **Lucy Hitcham:** Writing—original draft (equal).

## DECLARATION OF INTERESTS

R.J. has received funding for gambling research projects in the last 3 years from GREO Evidence Insights and the Academic Forum for the Study of Gambling. These funds are sourced from regulatory settlements levied by the Gambling Commission in lieu of penalties. L.H. is funded by the Engineering and Physical Sciences Research Council (EPSRC) on a PhD Studentship scholarship (EP/S023305/1).

## Data Availability

Data sharing is not applicable to this article as no new data were created or analyzed in this study.
